# Clinical significance of circulating tumor cells in predicating the outcomes of patients with colorectal cancer

**DOI:** 10.1016/j.clinsp.2022.100070

**Published:** 2022-09-07

**Authors:** Kehe Chen, Zhenxiang Chen, Mei Ou, Junping Wang, Xiao Huang, Yingying Wu, Wenhe Zhong, Jiao Yang, Jinging Huang, Min Huang, Deng Pan

**Affiliations:** Department of Medical Oncology, The People's Hospital of Guangxi Zhuang Autonomous Region, Nanning, China

**Keywords:** Colon cancer, Circulating tumour cells, Relapse, Metastasis, Overall survival

## Abstract

**Background:**

Relapse and metastasis of patients with Colorectal Cancer (CRC) is the major obstacle to the long-term life of patients. Its mechanisms remain defined.

**Methods:**

A total of 48 CRC patients were enrolled and 68 samples were obtained from the peripheral blood of patients before or after treatments in this study. Twenty non-cancer patients were also detected as a negative control. Circulating Tumor Cells (CTCs), including Epithelial CTCs (eCTCs), Mesenchymal (MCTCs), and epithelial/mesenchymal mixed phenotypes (mixed CTCs), were identified by CanPatrol^TM^ CTC enrichment and RNA in situ hybridization. The relationship between CTCs number and Progression-Free Survival (PFS) or Overall Survival (OS) was evaluated.

**Results:**

Thirty-four of 48 patients (70.8%) were found to have positive CTCs. Total CTCs and MCTCs in the post-treatment had a significant correlation PFS and OS. When total CTCs or MCTCs in 5 mL blood of patients were more than 6 CTCs or 5 MCTCs, PFS of the patients was significantly shorter (p < 0.05) than that in patients with less than 6 CTCs or 5 MCTCs. The patients with > 5 CTCs count changes were found to exhibit poor PFS and OS rates (p < 0.05).

**Conclusion:**

Total CTCs and MCTCs number detection in patients with colorectal cancer was very useful biomarker for predicting the prognosis of patients. Higher CTCs or MCTCs had poorer PFS and OS rates.

## HIGHLIGHTS

• Total CTCs and MCTCs in post-treatment is strongly relative to PFS and OS of patients.
• The PFS of >6 CTCs or 5 MCTCs/5 mL blood was shorter than that in ≤ 6 CTCs or 5 MCTCs.
• Detection of CTCs in CRC patients is a critical biomarker for their prognosis.

## Introduction

Worldwide, Colorectal Cancer (CRC) is the fourth most lethal cancer after lung, stomach, and liver cancer.^1^ Its incidence ranks second cancer in women and third cancer in men.^2^ Bad eating habits, smoking, insufficient physical exercise, and obesity are the most common risk factors for the occurrence of CRC.^3^ In addition, the patients with hereditary nonpolyposis colorectal cancer (HNPCC^4^ or Lynch syndrome) and inflammatory bowel disease^5^ also have a risk for this disease. CRC treatments include surgery, chemotherapy, radiotherapy, and immunotherapy, which are very useful for those patients with mismatch repair deficiency and microsatellite instability.^6^^,^^7^

Although diagnosis and treatments of CRC had great progress in the past decades, its long-term survival rate is less than 60% or more low.^4^ The survival rate of CRC is directly related to the types and Tumor-Node-Metastasis (TNM stages) of CRC.^8^ In addition, the survival rate of CRC is also linked to specific gene expressions such as HSPA1A and RBM3.^9^ Recent studies revealed that the presence of Circulation Tumor Cells (CTCs) in cancer patients was closely associated with the relapse and metastasis of cancer.^10^, ^11^, ^12^ CTCs are cells originally from a primary tumor and can become seeds for tumor growth in distant organs by blood circulation system.^13^^,^^14^ This mechanism was the reason for the recurrence and metastasis of many cancers such as breast cancer,^15^ lung cancer,^16^ gastric cancer,^17^ head and neck cancers,^18^, ^19^, ^20^ as well as resistance to anti-cancer treatment.

In contrast, CTC-based liquid biopsies have a few advantages over traditional method.^21^^,^^22^ First, it is a non-invasive and repeatable technique for monitoring metastasis, progress, and therapeutic efficiency of cancer.^23^ Second, cell resources are from the peripheral blood of the patients and are easily obtained in the solid tumor.^24^ Third, the CTC detections of solid tumors avoid surgery for evaluating metastasis of cancer.^25^ Therefore, monitoring changes of CTCs in cancer patients have a great clinical value in assessing diagnosis, therapy, relapse, metastasis, and prognosis of cancer patients. In our study, the authors aim to investigate the correlation between CTCs profiles and the clinical features of CRC.

## Materials and methods

### Subjects and samples

A total of 48 CRC and 20 patients without tumors as a control were enrolled in this study from January 2018 to October 2020. The diagnosis of these patients was confirmed by pathological and radiological findings from surgery or biopsy samples. TNM staging system for CRC patients followed the criteria of the American Joint Committee on Cancer (AJCC) 8th edition.^2^ Those patients outside of the studied hospital or without tumor tissue available were excluded from this study. All participants also must meet the following criteria: no active infection and normal liver, kidney, and cardiology functions. A total of 5 milliliters (mL) of whole blood samples from patients were collected before or after treatments. All clinical characteristics of patients were documented and identified. All patients also followed up for their PFS and Overall Survival (OS). PFS was calculated as the time from the initial treatment to disease progression. OS is the time from the initial treatment to death. Tumor burden were counted using clinical and imaging data like Computer Tomography (CT) or Magnetic Resonance Imaging (MRI).

The study protocol was reviewed and approved by the ethics committee of the people's hospital of Guangxi Zhuang autonomous region. Written informed consent has obtained from all patients before the study. All procedures performed in the studies were in accordance with the ethical standards of the 1964 Helsinki declaration and its later amendments or comparable ethical standards.

### Isolation and identification of CTCs

A filtration method was applied to isolate and identify CTCs following the previous technique.^26^^,^^27^ Briefly, blood samples were collected before and after treatment. Red blood cells were removed using a red blood cell lysis buffer (154 mM NH_4_Cl, 10 mM KHCO_3,_ and 0.1 mM EDTA) and incubated for 30 minutes at room temperature. The remaining cells were fixed containing 4% formaldehyde (Sigma, St. Louis, USA) with *Phosphate Buffered Saline (*PBS) solution for 5 minutes. Then the cells were transferred to the filtration tube with an 8 μM pore size filter membrane for the manifold vacuum plate and started to filtrate CTCs.

### Tri-color RNA in situ hybridization assay

The RNA-in situ hybridization^25^ technique based on the branched DNA (bDNA) signal amplification system^28^ was applied to characterize CTCs. Briefly, bDNA capture probe is used to bind > 30 specific nucleotide sequences called Label Extenders (LEs) of an interested gene, which LE probes bind preamplifiers. In turn, preamplifiers bind many amplifiers and the label probe, which are the Alexa Fluor 594 conjugated epithelial biomarkers EpCAM and CK8/18/19, Alexa Fluor 488 conjugated mesenchymal biomarkers vimentin and twist, and 4′,6-Diamidino-2-Phenylindole 9 (DAPI) staining. The results are that low up to 10 copies of original specific gene sequences can be accurately amplified between ∼10,000 and 10,000,000 fold. In addition, these fluorescents labeled antibodies are easily observed and counted in a fluorescent microscope.

### Statistical analysis

Data were analyzed using statistic analyzing Excel and GraphPad Prism8 software. The χ2 test, *t*-test, and Kaplan-Meier analysis were performed to analyze the data; p < 0.05 was considered a significant difference.

## Results

### Patients and CTC characteristics

A total of 48 CRC patients and 20 free-tumor control patients were recruited for this study. The detailed patient characteristics are shown in Table 1. Their average age was 52.42 years old and the range was from 20‒80 years old, including 26 males and 22 females. The number of patients from the cecum, ascending colon, transverse colon, descending colon, and sigmoid colon were 3, 7, 5, 5, and 28, respectively. Stage II, III, and IV patient numbers were 28, 12, and 8, respectively. Low, moderate, and well differentiation patients were 6, 35, and 7, respectively. Twenty patients had metastatic disease, including 13 cases of lympho-node and 8 cases of organ metastasis.

**Table 1:** The clinical Characteristics of colorectal patients and controls.

		**Patients number**		**Control number**
**Characteristics**	**NO**	**%**	**NO**	**%**
Age (Year-old)				
Mean	52.42		47.5	
Range	22‒80		32‒74	
Gender				
Female	26	54	10	50
Male	22	46	10	50
Tumor Location				
Cecum	3	6.3	N/A	
Ascending colon	7	14.6		
Transverse colon	5	10.4		
Decending colon	5	10.4		
Sigmoid colon	28	58.3		
Stage				
II	28	58.3	N/A	
III	12	25.0		
IV	8	16.7		
Differentiation				
Low	6	13	N/A	
Moderate	35	73		
Well	7	14		
Metastasis				
Lymphonode	13	27.0		
Organ	7	14.6		

### The association of CTCs number with the clinical characteristics

**Fig. 1:**
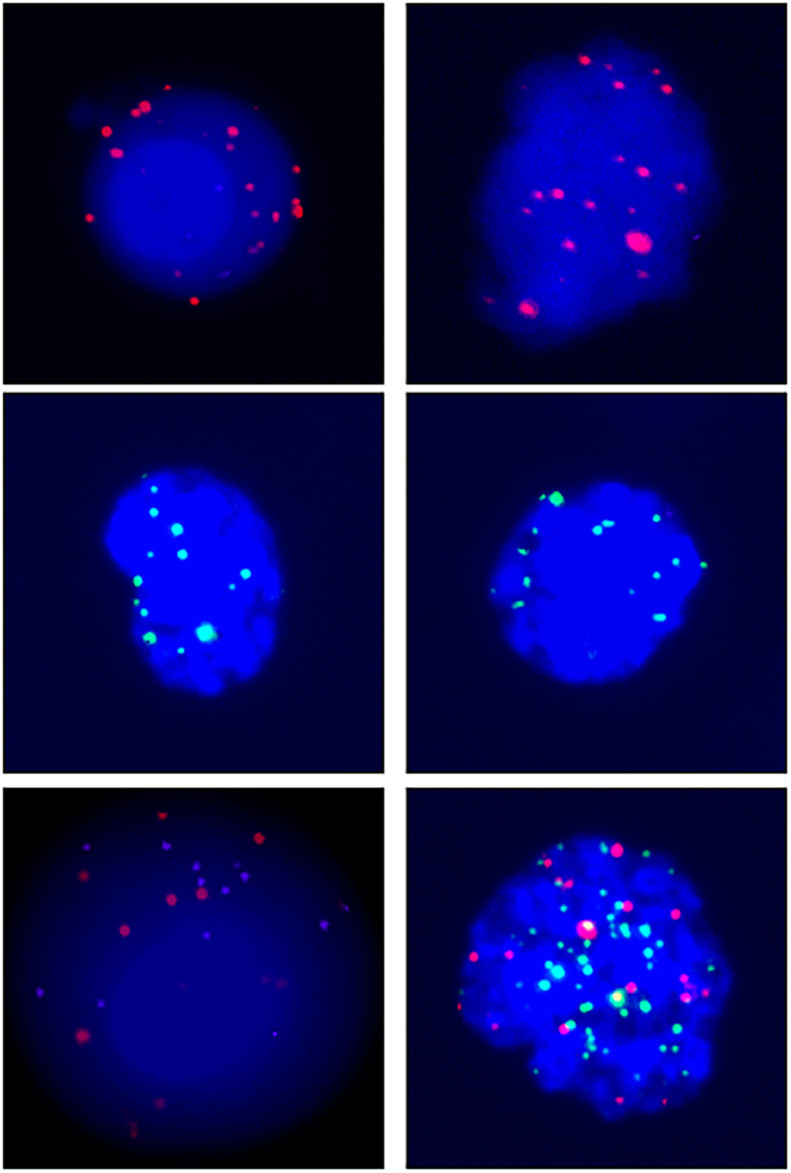
Images of CTCs. Up, middle, and low panels show the pictures of epithelial, mesenchymal,and mixed CTCs. Epithelial CTCs were stained with only Alexa Fluor 594 labeled epithelial markers EpCAM and CK8/18/19. Mesenchymal CTCs were stained with Alexa Fluor 488 labeled Vimentin and Twist. Pictures were taken by 40 ×  magnification in immunofluorescence microscope.

**Fig. 2:**
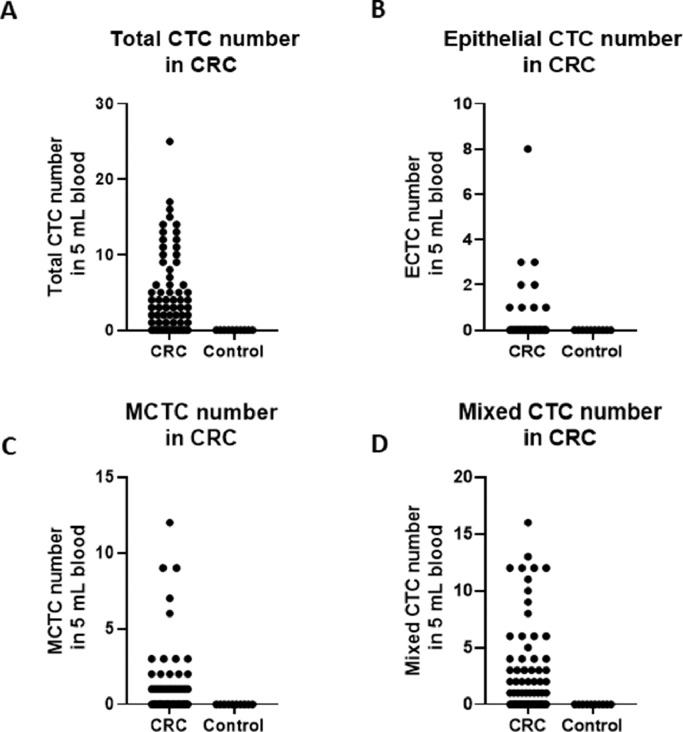
Comparsion of CTCs and subtypes in CRC patients and control patients. (A) Total CTC number comparison; (B) Epithelial CTC number comparison; (C) Mixed CTC number comparison; (D) MCTC number comparison. CTCs, Circulating Tumor Cells; MCTC, Mesenchyal Circulating Tumor Cell.

A total of 68 blood samples were collected before or after treatment, respectively. CTCs images are shown in Fig. 1. Based on the CTC surface mRNA markers, the CTCs were classified into three phenotypes: epithelial, mixed phenotypic, and mesenchymal CTCs. All 48 patients had obtained CTC assessment. The CTCs were detected in 34 of the 48 patients (70.8 %) with an average count of 6.76 CTCs (range 0‒17 per 5 mL blood). The results showed that there were 14 negative cases (29.2%) and 34 positive cases (70.8%). The authors also counted the total MCTCs, including MCTCs plus mixed CTCs cells, which account for 35.4% (17 cases). Their profiles are shown in Fig. 2. Compared with non-tumor control, total CTCs, epithelial CTCs (eCTCs), MCTCs, and mixed CTCs in CRC patients were significantly higher than in control cases. The authors compared negative and positive CTCs with gender, T-staging, N-staging, smoking history, and tumor locations of patients (Table 2). There were no significant differences among CTCs of patients in gender, smoking history, and tumor locations. However, CTCs positive cases in T3, T4, and above N2b staging were significantly higher than that in T2 and less N2b staging. These results indicated that there was a higher CTC positive rate in patients with CRC patients with advanced T-stages.

**Table 2:** The clinical parameters associated with baseline CTCs in the CRC patients before treatments (Fisher's exact test and Pearson Chi-Square test).

**Variants**	**0 CTC (%)(*n* = 14)**	**≥ 1 CTC (%)(*n* = 34)**	***p*-value**
Gender			15 (31.4)
Female	7 (14.6)	19 (39.4)	0.758
Male	7 (14.6)		
T-staging			0.03
T2	12 (25)	16 (33.3)	
T3	2 (8.3)	10 (20.8)	
T4	0 (0.0)	8 (16.7)	
N staging			0.02
< N2b	13 (27.1)	10 (20.8)	
≥ N2b	2 (4.2)	23 (47.8)	
Smoking History			0.63
Smoking	4(8.3)	4(8.3)	
Non-smoking	10 (20.9)	30 (62.5)	
Tumor location			0.89
Cecum	1 (2.1)	2 (4.2)	
Ascending Colon	2 (4.2)	5 (10.4)	
Transverse Colon	2 (4.2)	3 (6.3)	
Decending Colon	2 (4.2)	3 (6.3)	
Sigmoid Colon	7 (14.6)	21 (43.8)	

CTCs, Circulating Tumor Cells; n, Case number.

### Relationships between CTCs number and the outcomes of patients after treatment

**Fig. 3:**
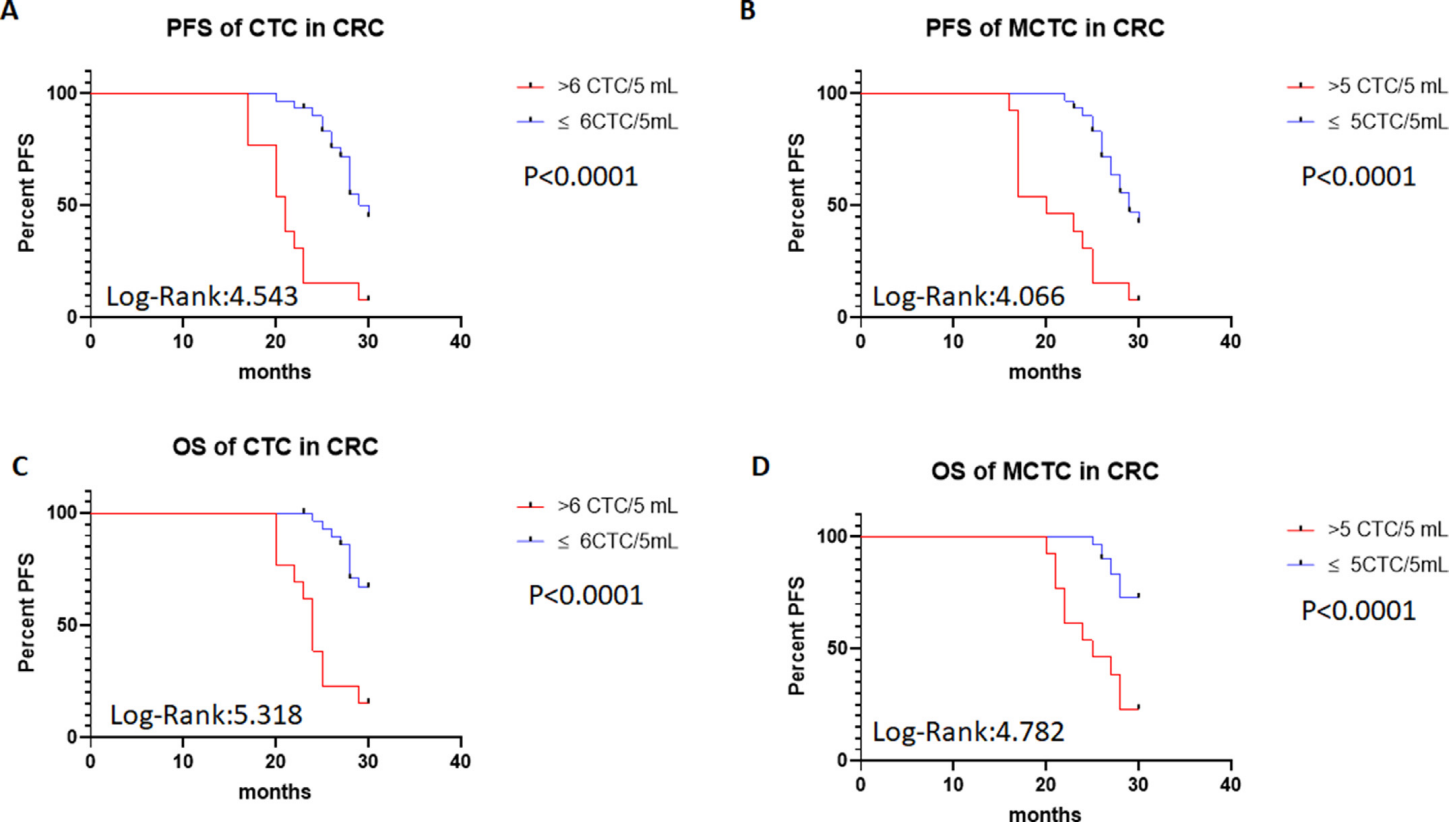
PFS of patients with CTCs and MCTC by Kaplan-Meier curves at diagnosis. (A) PFS in > 6 CTCs vs. ≤ 6 CTCs of CRC patients; (B) PFS in > 5 MCTCs vs. ≤ 5 MCTCs of CRC patients; (C) Comparison of OS > 6 CTCs vs. ≤ 6 CTCs of CRC patients; (D) Comparison of OS > 5 MCTCs vs. ≤5 MCTCs of CRC patients. CTCs, Circulating Tumor Cells; MCTC, Mesenchyal Circulating Tumor Cell; PFS, Progression-Free Survival; OS, Overall Durvival.

To investigate the roles of CTC numbers in the outcomes of CRC patients, the authors traced the patient's outcomes up to 30 months after treatments and recorded their PFS and OS times. PFS was defined as the time from treatment to the disease's progress. OS was defined as the time from treatments to the patient's death. The authors also compared the PFS and OS of patients using a cut-off value > 6 total CTCs or 5 MCTCs. The survival analysis was performed with Kaplan-Meier curves. The results are shown in Fig. 3. The authors found that when total CTCs were > 6 or MCTCs were > 5 MCTCs in 5 mL peripheral blood, PFS of the CRC patients in total > 6 CTCs or > 5 MCTCs group was significantly shorter than those patients with ≤ 6 CTCs or ≤ 5 MCTCs (p < 0.0001 and < 0.0001, Log-rank 4.543 and 4.066, respectively; Fig. 3A‒B). Similarly, when the presence of > 6 CTCs or > 5 MCTCs in 5 mL peripheral blood from patients was found, the OS of the CRC patients also displayed a poorer than those patients with ≤ 6 CTCs or ≤ 5 MCTCs (p < 0.0001 and p < 0.0001, Log-rank 5.318 and Log-rank 4.782, respectively; Fig. 3C‒D). These results revealed that CTCs monitored in the patients were a very useful biomarker for predicting the outcomes of CRC patients. Moreover, the number of metastatic lesions was also associated with progression-free survival, patients with > 1 metastatic lesion were found to exhibit a poorer PFS than those with ≤ 1 metastatic lesion. The authors investigated the prognostic potential of the changes in a number of CTC after treatment by Kaplan–Meier survival analyses for PFS and OS. The patients with ≥ 4 CTC number changed after treatment were found to exhibit a poorer PFS and OS than those with < 4 CTC number changed after treatments (data not shown). These results indicated that CTCs detection in CRC patients was a critical biomarker in predicting the prognosis of patients.

## Discussion

Recently, applications of liquid biopsy techniques like CanPatrol™ CTC enrichment have greatly enhanced the clinical judgment of diagnosis, treatments, and outcomes.^29^, ^30^, ^31^, ^32^ Liquid biopsy has many advantages over the traditional methods for precision medicine and early detection of diseases, including non-invasive ways and easily obtained samples like blood and urine. In the current study, the authors used CanPatrol™ CTC-enrichment and RNA-ISH assay to monitor CTCs changes in CRC patients. RNA-ISH assay has a few advantages over techniques for CTC detections: first, it can detect low to 10 copies of the target gene; second, RNA-ISH can accurately and precisely amplify the target gene by ∼10000‒10000000 fold;^28^ third, RNA-ISH combined with a fluorescent microscope or flow cytometry to quantify target gene expression.^33^^,^^34^ Recent literature have reported that CTC was strongly related to the progress, relapse, and metastasis of some cancers.^35^, ^36^, ^37^ CTCs have been recommended as a cancer hallmark by the American Society of Clinical Oncology for the ability to detect lesions in early stages in patients without cancer symptoms 2007.^38^^,^^39^ Here, the present results confirmed that CTC can be used as a useful biomarker for the prognosis of tumors and explored the clinical significance of CTCs count in CRC patients.

The present data showed that there were no significant differences between negative and positive CTCs in gender, smoking history, and tumor locations after treatment. However, the authors found that when numbers of total CTCs and MCTCs were higher than 6 or 5 in 5 mL peripheral blood, PFS and OS of the CRC patients were dramatically shorter than those patients with ≤ 6 CTCs or ≤ 5 MCTCs per 5 mL blood. The clinical significance of CTC in different cancer patients was extensively investigated^10^^,^^11^^,^^40^^,^^41^ High CTC number was especially relevant to relapse and metastasis of advanced cancer patients, which are the major reasons for the high fatality rate. The mechanism of relapse and metastasis in cancer patients is very complex. The presence of CTCs is one of these reasons. The study showed that aberrant activation of the Epithelail-Mesenchymal Transition (EMT) of CTCs was the key mechanism of cancer metastasis.^42^ The present data confirmed that total high CTCs and MCTCs in the CRC patients had shorter PFS and OS. This result indicated that CTC was a very useful biomarker for monitoring the progress of CRC patients.

Previous studies revealed that CTC detection may help to assess the high-risk patient selection for adjuvant chemotherapy and guide the prognosis.^43^^,^^44^ For the CRC patients, it was reported that Tumor-Derived Extracellular Vesicles (tdEV), Endothelium-Derived Extracellular Vesicles (edEVs), and CTCs detection were more powerful for predicting OS of the CRC patients.^45^ Here, the authors found that the combination of total CTCs and MCTCs was a more effective t to monitoring the survival status of CRC patients than the alone number. In addition, the authors found that the sigmoid colon was the major site for the occurrence of CRC Cancer. However, the size of this study cohort was 48 patients and 20 control cases. This study size was limited. In addition, the follow-up time was 30 months, which limited PFS and OS times. Therefore, large size and longer time follow-up studies will be needed to confirm the current data.

In conclusion, Total CTCs and MCTCs number detection of the CPC patients was a very useful biomarker for predicting the prognosis of patients; > 6 CTCs or 5 MCTCs in 5 mL peripheral blood had a shorter PFS and OS. Therefore, CTCs and MCTCs number detection provided a new therapeutic strategy for CRC patients.

### Ethics approval and informed consent

The study was approved by the ethics committee of the people's hospital of Guangxi Zhuang autonomous region (Approval n° KY-LW-2017-1). Written informed consent has obtained from all patients before the study. All procedures performed in the studies were in accordance with the ethical standards of the 1964 Helsinki declaration and its later amendments or comparable ethical standards.

## Conflicts of interest

The authors declare no conflicts of interest.
